# Complement factors C1q, C3 and C5 in brain and serum of mice with cerebral malaria

**DOI:** 10.1186/1475-2875-7-207

**Published:** 2008-10-10

**Authors:** Peter Lackner, Christian Hametner, Ronny Beer, Christoph Burger, Gregor Broessner, Raimund Helbok, Cornelia Speth, Erich Schmutzhard

**Affiliations:** 1 Department of Neurology, Innsbruck Medical University, Innsbruck, Austria; 2 Division of Hygiene and Medical Microbiology, Innsbruck Medical University, Innsbruck, Austria

## Abstract

**Background:**

The patho-mechanisms leading to brain damage due to cerebral malaria (CM) are yet not fully understood. Immune-mediated and ischaemic mechanisms have been implicated. The role of complement factors C1q, C3 and C5 for the pathogenesis of CM were investigated in this study.

**Methods:**

C57BL/6J mice were infected with *Plasmodium berghei ANKA *blood stages. The clinical severity of the disease was assessed by a battery of 40 standardized tests for evaluating neurological functions in mice. Brain homogenates and sera of mice with CM, infected animals without CM and non-infected control animals were analyzed for C1q, C3 and C5 up-regulation by Western blotting.

**Results:**

Densitometric analysis of Western blots of brain homogenates yielded statistically significant differences in the levels of C1q and C5 in the analyzed groups. Correlation analysis showed a statistically significant association of C1q and C5 levels with the clinical severity of the disease. More severely affected animals showed higher levels of C1q and C5. No differences in complement levels were observed between frontal and caudal parts of the brain. Densitometric analysis of Western blot of sera yielded statistically lower levels of C1q in infected animals without CM compared to animals of the control group.

**Conclusion:**

The current study provides direct evidence for up-regulation of complement factors C1q and C5 in the brains of animals with CM. Local complement up-regulation is a possible mechanism for brain damage in experimental cerebral malaria.

## Background

Cerebral malaria (CM) is a major cause of morbidity and mortality of *Plasmodium falciparum *malaria. It presents as a diffuse encephalopathy with alteration of consciousness, ranging from drowsiness to deep coma and is frequently accompanied by seizures [1]. Mortality is high and neurological sequelae are observed in approximately 10% of the survivors [2]. The pathophysiological mechanisms of CM are not yet fully understood. Most researchers agree that the immune response of the host is a critical factor in the pathogenesis of CM. Different aspects have been studied and in particular pro-inflammatory cytokines and activated T-lymphocytes have been shown to be related to the development of CM [3,4]. One potent stimulator of inflammation is the complement system. It consists of about 30 fluid-phase and cell-membrane proteins and is important not only to recognize but also kill pathogens such as bacteria, virus infected cells and parasites, while preserving normal 'self' cells. Complement can be activated by two distinct routes, the classical and the alternative pathway. The classical pathway is activated primarily by the interaction of C1q with immune complexes (antibody-antigen). The alternative pathway is triggered directly on pathogen surfaces and leads to the deposition of C3 fragments (opsonins) on the target cells. The ultimate goal for the activation of the complement system is the formation of the membrane attack complex which is initiated by proteolytical cleavage of C5 and disrupts the phospholipid bilayer to lyse the target cell [5].

In addition, the small complement fragments C3a, C4a and C5a, the so-called anaphylatoxins act on specific receptors to produce local inflammatory responses. They are released in the fluid phase during complement activation after enzymatic cleavage of C3 and C5. These factors are acting directly on blood vessels, stimulating an increase in blood flow, increasing vascular permeability and increase the binding of phagocytes to endothelial cells [5]. C5a also activates mast cells to release mediators such as histamine and TNF-alpha that contribute to the inflammatory response [6]. While data on complement factors in neuroinflammation in CM is still limited, some reports show the important role of complement factors in systemic inflammatory response to murine malaria infection [7,8].

Besides its potential to induce a marked local inflammation the pro-apoptotic capacity of C5a in neurons is of interest [9,10] since apoptosis has been shown to be an important neuropathological feature of murine CM [11,12]. A further focus in the pathophysiology of murine CM is the integrity of the blood-brain barrier (BBB) [13]. BBB dysfunction may allow the influx of cytokines, malaria antigens and complement into the brain. C1q has been reported to have a role in BBB breakdown in an experimental BBB disintegration model [14]. C1q is produced by microglia and astrocytes. Activation of both cell types precedes clinical symptoms of CM [15,16]. Importantly, increased gene-expression of C1q is found in the brains of CM susceptible mice, as compared to animals resistant to CM, in *Plasmodium berghei *infection [17]. In addition, C1q, beside anaphylatoxins, is able to trigger a proinflammatory immune response by inducing cytokines and chemokines and activation of neutrophils and eosinophils [18].

Although it has been shown that genes of identified complement-related function are induced during murine CM [19], data on protein expression of complement factors in the murine brain are missing. The current study was conducted to analyze complement factors C1q, C3 and C5 – catalyzing crucial steps in the complement cascade – in the brains and sera of mice infected with *Plasmodium berghei ANKA *– a well-established model of cerebral malaria.

## Methods

### Animals and sample preparation

Nineteen 6 to 8 weeks old C57BL/6J mice (Charles River, Sulzfeld, Germany) were used for this study. Fifteen animals were infected intraperitoneally with 5.10^6 ^parasitized red blood cells of a homologue donor, which had been infected with frozen polyclonal stocks of *P. berghei ANKA*. Parasitaemia was monitored every other day. The clinical severity of the disease was assessed by the SHIRPA-score primary screen to discriminate different levels of severity in murine CM [20]. The primary screen comprises a battery of 40 simple tests for evaluating neuromuscular, spinocerebellar, sensory, neuropsychiatric and autonomic functions in mice by observational assessment. The scoring starts with the evaluation of undisturbed behavior in a viewing jar. Subsequently the mouse is transferred to an arena for observation of motor behaviour. Afterwards a sequence of manipulations using tail suspension is performed and visual acuity, grip strength, body tone and reflexes are recorded. Finally autonomous functions like skin color and heart rate are assessed in supine restraint and the procedure is ended with measurement of rectal body temperature. All test values are then summed up and the cumulative SHIRPA-score is calculated as described previously [20]. Healthy mice show a value of about 30, while moribund CM animals show values of about 10. Animals at different clinical stages of the disease were killed and further processed for Western blot analysis. Four non-infected C57BL/6J mice (CNT) and three infected animals that did not develop CM (NCM) served as controls. All animals were given a lethal dose of 0.5 ml (25 mg/ml) thiopental (Biochemie, Kundl, Austria) intraperitoneally. Deeply anesthetized mice (total n = 19, CM n = 12, NCM n = 3, CNT n = 4) were transcardially perfused with PBS for 2 min with a pressure controlled syringe pump (Fresenius-Kabi, Bad Homburg, Germany). Mouse serum was harvested immediately before perfusion by puncture of the right cardiac ventricle. Animal studies conformed to the Austrian guidelines for the care and use of laboratory animals and were approved by the Austrian Government.

### Western Blot

Pivotal complement factors C1q, C3 and C5 were analyzed in protein balanced brain homogenates and sera of mice with different clinical levels of severity of CM (CM, n = 12), infected animals without neurological affection (NCM, n = 3) and non-infected control animals (CNT, n = 4). Samples of frontal (cerebrum) and caudal (brain stem and cerebellum) parts of the brain were processed separately. The microdissected tissue was homogenized in ice-cold buffer (pH 7.5) containing 50 mM Tris-Cl, 5 mM EDTA, 50 mM NaCl, 5 mM DTT, 0.1% Np-40, 50 mM NaF, 1 mM PMSF, 1 mM Na_3_VO_4 _plus a protease inhibitor cocktail (Roche, Mannheim, Germany) and centrifuged at 18500 g for 20 min at 4°C. Protein-balanced samples were analyzed using standard techniques. Antibodies were diluted in blocking solution (2% milk powder in PBS). Bottom and top parts of the blots were probed with monoclonal antibodies, C1q-a (M-120, Santa Cruz Biotechnology, Santa Cruz, USA; 1:1,000 in blocking solution) and C5 (Dako, Glostrup, Denmark; 1:2,000 in blocking solution) overnight at 4°C. To control and correct for equal loading, the middle part of each blot was probed for alpha-tubulin (Sigma, St. Louis, USA; 1:40,000 in blocking solution) overnight at 4°C. Antibody binding was visualized using enhanced chemoluminescence reagents (Lumiglo™; Boston, USA, Cell Signaling). After film development, antibodies were stripped in stripping solution (pH 8.8) containing 1 M Tris, 10% SDS, 0.5% 2-mercaptoethanol in a water bath at 70°C for 20 minutes. After rinsing in PBS-T (PBS containing 0.1% Triton-X 100) for 45 minutes the top part of the blot was probed with a polyclonal antibody C3 (H-300, Santa Cruz Biotechnology, 1:1000 in blocking solution), which detects full length and the alpha-subunit (110 kD) of C3. Antibody binding was visualized using enhanced chemoluminescence reagents (Lumiglo™; Cell Signaling).

### Statistical methods

Densitometric values of complement factors C1q, C3 and C5 of frontal and caudal parts of the brain were compared using Wilcoxon signed rank sum test. Since there was no statistical difference between the data of the respective brain areas, the mean values between frontal and caudal parts of the brain were calculated and used for further analyses. Kruskal-Wallis test and Dunn's post test were used to analyze complement levels in the different disease groups. Spearman's rho was calculated between the cumulative SHIRPA-score and the densitometric values of C1q, C3 and C5. Calculations were done using Insightful S-Plus 6.2 (Insightful Corporation, Seattle, WA, USA), graphs were drawn with GraphPad Prism 5.00 (GraphPad Software, San Diego, USA).

## Results

Twelve of the 15 infected animals developed signs of CM between day-5 and day-7 post-infection (day-5 n = 2; day-6 n = 7; day-7 n = 3) with low levels of parasitaemia between 5% and 15% (CM group). Animals with CM were divided into three CM severity groups for further analysis according to the SHIRPA-score (mild: CM1 = SHIRPA-score >25; moderate: CM2 = SHIRPA-score 15–25; severe: CM3 = SHIRPA-score < 15). The mean body temperature was 37.6 (CM1), 31.9 (CM2) and 28.7 (CM3) degree Celsius in the respective groups. Three animals survived the CM vulnerable period and were killed at day-11 post-infection. Since these mice did not show neurological signs they served as an additional control group (NCM group). Parasitaemia levels in the early course of the infection were highly similar between the two groups. Comparing the densitometric values of C1q, C3 and C5 in homogenates from frontal (cerebrum) and caudal (brain stem and cerebellum) parts of the brain in mice with CM did not yield significant differences.

### C1q is significantly higher in brain homogenates of mice with CM

**Figure 1:**
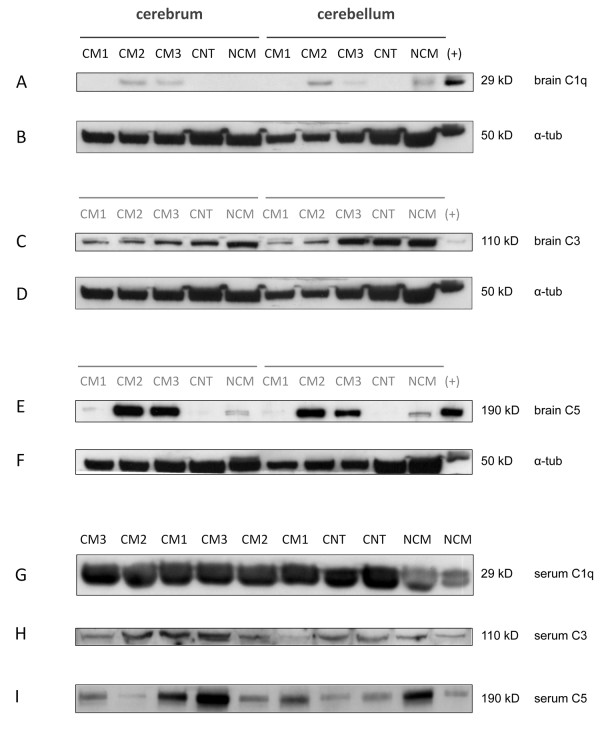
**Representative Western blot experiments of complement factors C1q (A), C3 (C) and C5 (E) in brains and in sera (G-I) of animals with different clinical levels of severity of cerebral malaria (CM1-3), infected animals without CM (NCM) and non infected control animals (CNT).** Alpha-tubulin (B, D, F). (+) = positive control.

**Figure 2:**
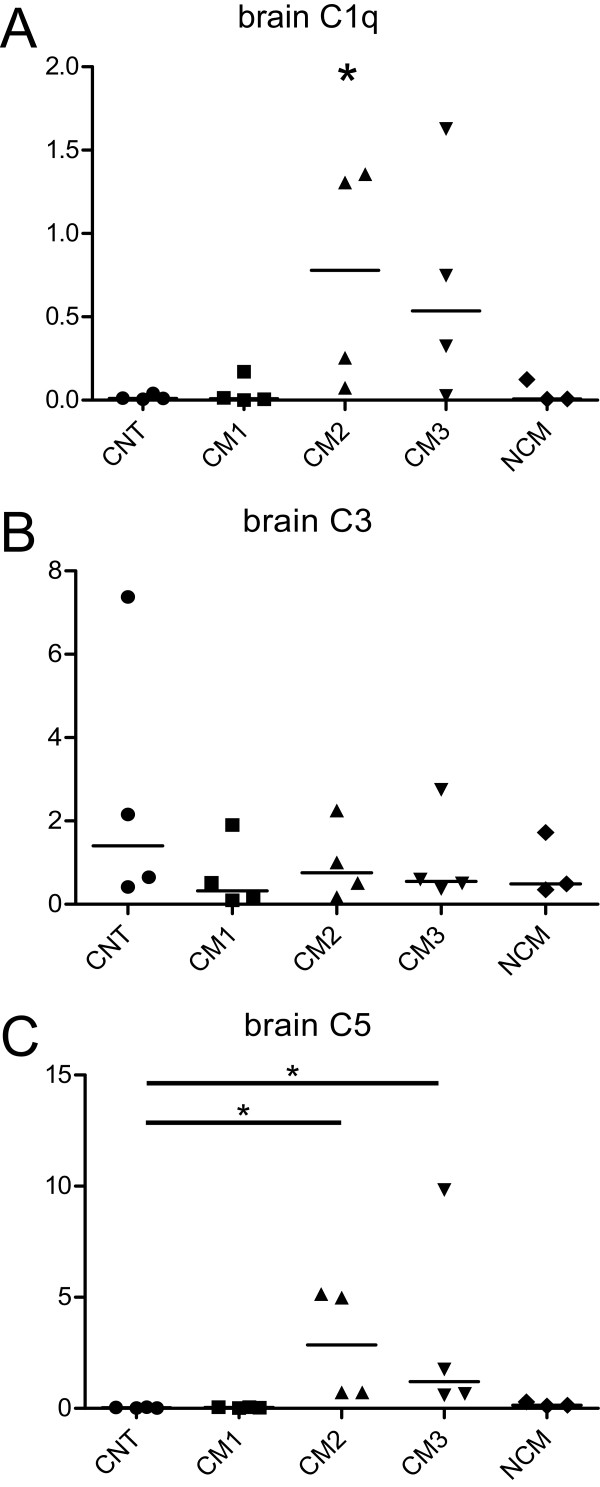
**Densitometric analysis of Western blots of complement factors C1q (A), C3 (B) and C5 (C) in brain homogenates of animals with different clinical levels of disease severity (CM1-3, n = 12), infected animals without CM (NCM, n = 3) and non-infected control animals (CNT, n = 4).** *, p < 0.05.

**Figure 3:**
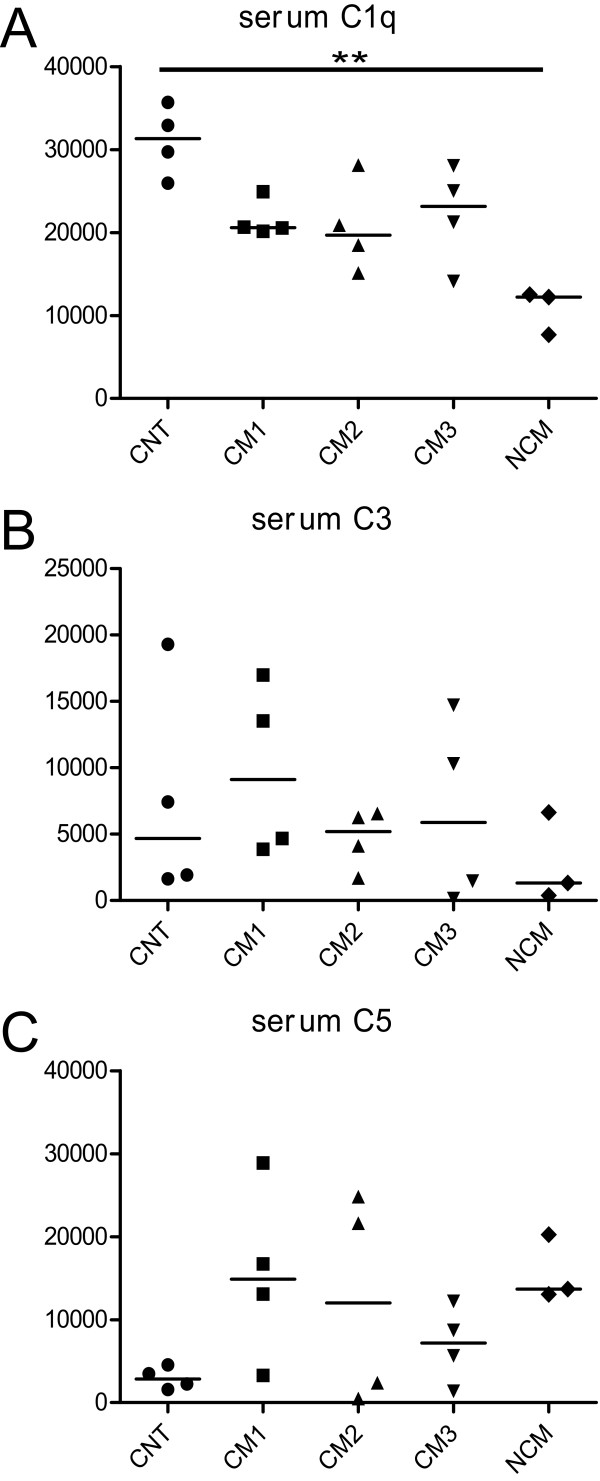
**Densitometric analysis of Western blots of complement factors C1q (A), C3 (B) and C5 (C) in sera of animals with different clinical levels of severity of cerebral malaria (CM1-3, n = 12), infected animals without CM (NCM, n = 3) and non-infected control animals (CNT, n = 4).** **, p < 0.01.

**Figure 4:**
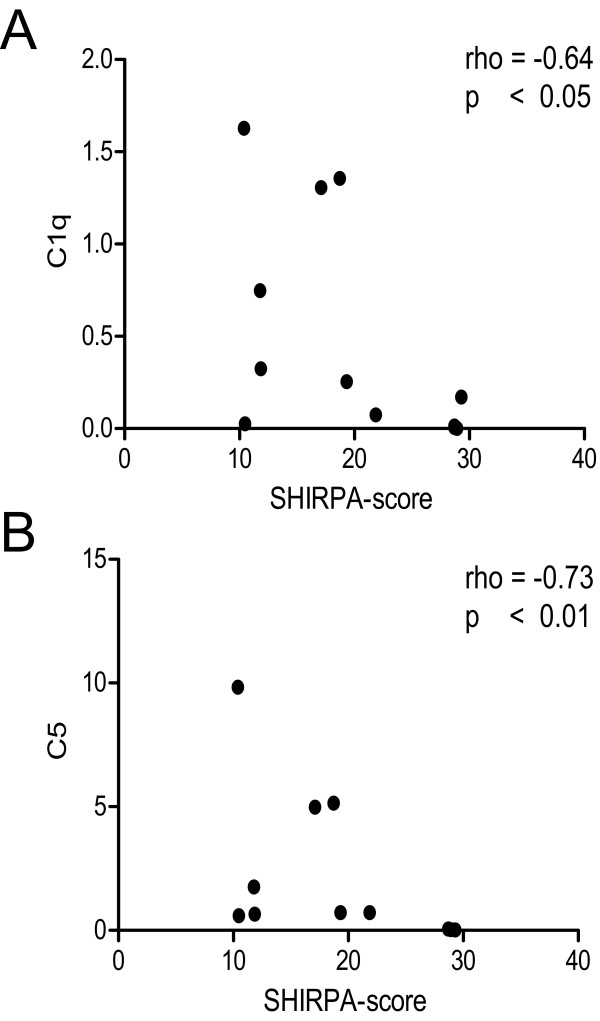
**Correlation analysis of densitometric values of C1q (A) and C5 (B) and the clinical severity of the disease (SHIRPA-score) in animals with CM (n = 12).** Spearman's rho and p-value are shown.

C1q levels in brain homogenates showed a statistically significant difference in the analyzed groups (Figures 1A and 2A; Kruskal-Wallis test; p < 0.05). Post-hoc analysis did not show significant differences between the respective groups of interest. In mice with CM, Spearmen's Rho indicated a significant correlation of C1q complement values in brain homogenates and the clinical severity score (Figure 4A; rho = -0.64, p < 0.05), i.e. animals with more severe disease showed higher levels of brain C1q. Densitometric measures of C1q in sera of the studied animals revealed a statistically significant difference (Figure 3A). Post hoc analysis yielded significantly lower values in NCM animals compared to non infected control animals (Figures 1G and 3A).

### C3 levels are not altered during the course of murine CM

No significant alterations of complement levels of C3 in brains or sera were found in the analyzed groups (Figures 1C, 1H and 2B). In mice with CM there was no significant correlation between brain complement levels and the cumulative SHIRPA-score.

### C5 is significantly higher in brain homogenates of mice with CM

C5 levels in brain homogenates showed a statistically significant difference in the analyzed data groups (Figures 1E and 2C; Kruskal-Wallis test; p < 0.05). Post-hoc analysis showed significantly higher levels of C5 in animals with moderate (CM2) and severe CM (CM3) as compared to non-infected control animals (CNT). In mice with CM, Spearmen's rho indicated a significant correlation of C5 complement values in brain homogenates and the clinical severity score (Figure 4B; rho = -0.73, p < 0.01), i.e. animals with more severe disease showed higher levels of brain C5. Densitometric measures of C5 in sera of the studied animals revealed no statistically significant differences (Figure 3C).

## Discussion

In the present study, the dynamics of complement factors C1q, C3 and C5 in the brains and sera of mice with malaria were investigated. Statistically significant direct correlations of C1q and C5 levels in brains of CM animals with disease severity were found. In sera of infected animals without CNS involvement C1q levels were significantly lower than in non infected controls.

Only limited data on the regulation of complement factors in the brain during experimental CM exists. In a recent observation by Delahaye *et al *gene expression of C1q was increased in the brains of CM susceptible mice, compared to resistant ones. In the present study, protein levels of C1q were elevated in animals with severe CM. This could be contributed to increased local production of C1q. Pro-inflammatory cytokines (in particular TNF-alpha and IFN-gamma) are capable to induce expression of complement factors in brain parenchymal cells in vitro [21]. Gene-levels of pro-inflammatory cytokines have been shown to be up-regulated in brain tissue during CM [22] and recently Interferon responsive mechanisms were reported to be of critical importance to the susceptibility to experimental CM [19]. Further, microglia is the most abundant source of C1q in the brain [23] and microglia induction has been shown to be a feature of the early course of murine CM [16]. Noteworthily, the blood brain barrier is known to be impaired in murine CM and systemic leakage of C1q into the brain could also lead to the elevated C1q levels in brain homogenates. However, the normal or even reduced levels of C1q in sera of mice with CM taken together with the reported increase in C1q gene transcription during CM might indicate local production of C1q. These findings warrant further studies on the exact source of complement production in the murine brain during CM.

This is of importance since the complement system, when activated at an inappropriate site and/or to an inappropriate extent, is remarkably effective in damaging host tissues thereby causing pathological changes, as seen in degenerative and inflammatory disorders of the central nervous system [24]. To avoid damage by complement, host cells are usually protected by a battery of regulatory molecules, which inhibit assembly of the membrane attack complex and subsequent lysis of the host cell. However, it has been shown that C1q binds specifically to the membrane of neurons and leads to the activation of the classical pathway in an antibody-independent manner [25]. Therefore, one can speculate that direct damage to neurons by C1q mediated induction of the classical complement pathway with consecutive cell lysis might contribute to neuropathological changes seen in murine CM [26].

Another important pathophysiological mechanism of brain damage in murine CM is neuronal apoptosis [11,12]. It has been shown that neurons have the capability of expressing the C5a receptor which in turn can induce apoptosis after binding to its ligand C5a [10]. Importantly, the C5a receptor is up-regulated in different inflammatory diseases of the central nervous system [27], hence the marked increase of C5 in the present study could represent another source of neurodegenerative potential in murine CM. Interestingly, a previous study showed that apoptosis in the murine brain during CM was mainly observed in neurons and oligodendrocytes and to a much lesser extent in astrocytes and microglia [11]. The latter cells express surface as well as soluble inhibitors of complement [5] thereby rendering them less susceptible to C5a induced apoptosis. Indeed oral administration of C5a receptor antagonists reduces neuronal apoptosis and protects rats from 3-nitropropionic acid (3-NP)-induced Huntington's disease [28]. Furthermore, C5 deficiency and C5a or C5aR blockade protects mice infected with *Plasmodium berghei ANKA *against cerebral malaria [29]. Therefore, such agents represent promising candidates for adjunctive neuroprotective/immunomodulatory treatment of CM.

C5a is a potent anaphylatoxin which is known to induce the production of proinflammatory cytokines and histamine and to promote the expression of adhesion molecules on endothelial cells [6,30,31]. Histamine has recently been shown to be of critical importance to the neuropathology of CM and histamin-decarboxylase deficient mice were protected from CM [32]. Further, ICAM-1 is upregulated during murine CM and leukocyte sequestration to the brain endothelia is also linked to neuropathology [4,33,34]. It should be noted that in the current study, due to its central role to the complement cascade, full length C5 was investigated, hence, the implication of its breakdown product C5a in murine CM remains to be resolved.

Another important finding of this study is the lack of spatial differences in the levels of complement factors C1q, C3 and C5 between the frontal (cerebrum) and caudal parts (brain stem and cerebellum) of the brain. This is of particular interest since these findings are in contrast to previous observations by our group and others in which the brain stem was the primary focus of neuronal apoptosis and microhaemorrhages [11,20,35]. This indicates that factors other than complement activation are responsible for the selective vulnerability of this brain region.

The levels of C1q in sera of animals with CM were normal or even lower than those in CNT animals. Further, infected mice without neurological affection (NCM) showed significantly lower levels of C1q. This is in line with the observation that C1q hypocomplementemia along with increased levels of immune complexes were observed in the sera of humans with *P. falciparum *malaria [36]. In addition it was demonstrated that free haem inhibits the function of C1q [37]. Therefore, not only consumption of C1q due to immune complex elimination but also direct inhibition or down-regulation of C1q by free haem can be regarded as a contributor to the reduced C1q levels in NCM mice in the present study.

## Conclusion

In conclusion, the current data provides evidence for the induction of the complement cascade in the brains of mice with CM. This suggests a role of complement factors C1q and C5 in the neuropathology of CM and emphasizes the importance of the host immune response in this neuro-infectious disease. Further studies on complement are warranted in order to confirm the pathophysiological role of these factors in CM. This might open a new perspective for adjunctive therapeutical approaches targeting the innate immune system.

## Competing interests

The authors declare that they have no competing interests.

## Authors' contributions

PL, CH and CB performed all experiments and wrote the manuscript. RB, RH, GB, CS and ES wrote the manuscript and contributed to the analyses of the data. PL, CS and ES had the idea and helped with the interpretation of the data.

## References

[B1] Schmutzhard E, Gerstenbrand F (1984). Cerebral malaria in Tanzania. Its epidemiology, clinical symptoms and neurological long term sequelae in the light of 66 cases. Trans R Soc Trop Med Hyg.

[B2] Newton CR, Krishna S (1998). Severe falciparum malaria in children: current understanding of pathophysiology and supportive treatment. Pharmacol Ther.

[B3] Hansen DS, Siomos MA, Buckingham L, Scalzo AA, Schofield L (2003). Regulation of Murine Cerebral Malaria Pathogenesis by CD1d-Restricted NKT Cells and the Natural Killer Complex. Immunity.

[B4] Belnoue E, Kayibanda M, Vigario AM, Deschemin JC, Van Rooijen N, Viguier M, Snounou G, Renia L (2002). On the pathogenic role of brain-sequestered alphabeta CD8+ T cells in experimental cerebral malaria. J Immunol.

[B5] Speth C, Dierich MP, Gasque P (2002). Neuroinvasion by pathogens: a key role of the complement system. Mol Immunol.

[B6] DiScipio RG, Schraufstatter IU (2007). The role of the complement anaphylatoxins in the recruitment of eosinophils. Int Immunopharmacol.

[B7] Williams AI, Rosen FS, Hoff R (1975). Role of complement components in the susceptibility to *Plasmodium berghei *infection among inbred strains of mice. Ann Trop Med Parasitol.

[B8] Krettli AU, Nussenzweig V, Nussenzweig RS (1976). Complement alterations in rodent malaria. Am J Trop Med Hyg.

[B9] Farkas I, Baranyi L, Liposits ZS, Yamamoto T, Okada H (1998). Complement C5a anaphylatoxin fragment causes apoptosis in TGW neuroblastoma cells. Neuroscience.

[B10] Farkas I, Baranyi L, Takahashi M, Fukuda A, Liposits Z, Yamamoto T, Okada H (1998). A neuronal C5a receptor and an associated apoptotic signal transduction pathway. J Physiol.

[B11] Lackner P, Burger C, Pfaller K, Heussler V, Helbok R, Morandell M, Broessner G, Tannich E, Schmutzhard E, Beer R (2007). Apoptosis in experimental cerebral malaria: spatial profile of cleaved caspase-3 and ultrastructural alterations in different disease stages. Neuropathol Appl Neurobiol.

[B12] Wiese L, Kurtzhals JA, Penkowa M (2006). Neuronal apoptosis, metallothionein expression and proinflammatory responses during cerebral malaria in mice. Exp Neurol.

[B13] Medana IM, Turner GD (2006). Human cerebral malaria and the blood-brain barrier. Int J Parasitol.

[B14] Lynch NJ, Willis CL, Nolan CC, Roscher S, Fowler MJ, Weihe E, Ray DE, Schwaeble WJ (2004). Microglial activation and increased synthesis of complement component C1q precedes blood-brain barrier dysfunction in rats. Mol Immunol.

[B15] Medana IM, Chan-Ling T, Hunt NH (1996). Redistribution and degeneration of retinal astrocytes in experimental murine cerebral malaria: relationship to disruption of the blood-retinal barrier. Glia.

[B16] Medana IM, Hunt NH, Chan-Ling T (1997). Early activation of microglia in the pathogenesis of fatal murine cerebral malaria. Glia.

[B17] Delahaye NF, Coltel N, Puthier D, Flori L, Houlgatte R, Iraqi FA, Nguyen C, Grau GE, Rihet P (2006). Gene-expression profiling discriminates between cerebral malaria (CM)-susceptible mice and CM-resistant mice. J Infect Dis.

[B18] Lu JH, Teh BK, Wang L, Wang YN, Tan YS, Lai MC, Reid KB (2008). The classical and regulatory functions of c1q in immunity and autoimmunity. Cell Mol Immunol.

[B19] Lovegrove FE, Gharib SA, Patel SN, Hawkes CA, Kain KC, Liles WC (2007). Expression Microarray Analysis Implicates Apoptosis and Interferon-Responsive Mechanisms in Susceptibility to Experimental Cerebral Malaria. Am J Pathol.

[B20] Lackner P, Beer R, Heussler V, Goebel G, Rudzki D, Helbok R, Tannich E, Schmutzhard E (2006). Behavioural and histopathological alterations in mice with cerebral malaria. Neuropathol Appl Neurobiol.

[B21] Thomas A, Gasque P, Vaudry D, Gonzalez B, Fontaine M (2000). Expression of a complete and functional complement system by human neuronal cells in vitro. Int Immunol.

[B22] Jennings VM, Actor JK, Lal AA, Hunter RL (1997). Cytokine profile suggesting that murine cerebral malaria is an encephalitis. Infect Immun.

[B23] Morgan BP, Gasque P (1996). Expression of complement in the brain: role in health and disease. Immunology today.

[B24] Bonifati DM, Kishore U (2007). Role of complement in neurodegeneration and neuroinflammation. Mol Immunol.

[B25] Singhrao SK, Neal JW, Rushmere NK, Morgan BP, Gasque P (2000). Spontaneous classical pathway activation and deficiency of membrane regulators render human neurons susceptible to complement lysis. Am J Pathol.

[B26] Lackner P, Beer R, Helbok R, Broessner G, Engelhardt K, Brenneis C, Schmutzhard E, Pfaller K (2006). Scanning electron microscopy of the neuropathology of murine cerebral malaria. Malar J.

[B27] Gasque P, Singhrao SK, Neal JW, Gotze O, Morgan BP (1997). Expression of the receptor for complement C5a (CD88) is up-regulated on reactive astrocytes, microglia, and endothelial cells in the inflamed human central nervous system. Am J Pathol.

[B28] Woodruff TM, Crane JW, Proctor LM, Buller KM, Shek AB, de VK, Pollitt S, Williams HM, Shiels IA, Monk PN (2006). Therapeutic activity of C5a receptor antagonists in a rat model of neurodegeneration. FASEB J.

[B29] Patel SN, Berghout J, Lovegrove FE, Ayi K, Conroy A, Serghides L, Min-oo G, Gowda DC, Sarma JV, Rittirsch D (2008). C5 deficiency and C5a or C5aR blockade protects against cerebral malaria. J Exp Med.

[B30] Foreman KE, Glovsky MM, Warner RL, Horvath SJ, Ward PA (1996). Comparative effect of C3a and C5a on adhesion molecule expression on neutrophils and endothelial cells. Inflammation.

[B31] Hartmann K, Henz BM, Kruger-Krasagakes S, Kohl J, Burger R, Guhl S, Haase I, Lippert U, Zuberbier T (1997). C3a and C5a stimulate chemotaxis of human mast cells. Blood.

[B32] Beghdadi W, Porcherie A, Schneider BS, Dubayle D, Peronet R, Huerre M, Watanabe T, Ohtsu H, Louis J, Mecheri S (2008). Inhibition of histamine-mediated signaling confers significant protection against severe malaria in mouse models of disease. J Exp Med.

[B33] Favre N, Da Laperousaz C, Ryffel B, Weiss NA, Imhof BA, Rudin W, Lucas R, Piguet PF (1999). Role of ICAM-1 (CD54) in the development of murine cerebral malaria. Microbes Infect.

[B34] Engwerda CR, Mynott TL, Sawhney S, de Souza JB, Bickle QD, Kaye PM (2002). Locally up-regulated lymphotoxin alpha, not systemic tumor necrosis factor alpha, is the principle mediator of murine cerebral malaria. J Exp Med.

[B35] Ma N, Harding AJ, Pamphlett R, Chaudhri G, Hunt NH (1997). Increased c-fos expression in the brain during experimental murine cerebral malaria: possible association with neurologic complications. J Infect Dis.

[B36] Adam C, Geniteau M, Gougerot-Pocidalo M, Verroust P, Lebras J, Gibert C, Morel-Maroger L (1981). Cryoglobulins, circulating immune complexes, and complement activation in cerebral malaria. Infect Immun.

[B37] Dimitrov JD, Roumenina LT, Doltchinkova VR, Vassilev TL (2007). Iron Ions and Haeme Modulate the Binding Properties of Complement Subcomponent C1q and of Immunoglobulins. Scand J Immunol.

